# Differential Characteristics of the Metabolic Profiles and Microbial Community between Superior and Normal Grades of *Nongxiangxing-daqu*

**DOI:** 10.3390/foods13060914

**Published:** 2024-03-17

**Authors:** Xiaoge Hou, Ming Hui, Xiaoman Gu, Xin Shi, Chenming Fan, Junfei Wang, Xuesi Li, Chunmei Pan, Ruifang Li

**Affiliations:** 1 School of Biological Engineering, Henan University of Technology, Zhengzhou 450001, China; xiaogeh@126.com (X.H.); codon@stu.haut.edu.cn (C.F.); 2 Engineering Research Center of Henan Liquor Brewing Special Grains Development and Application, Henan University of Animal Husbandry and Economy, Zhengzhou 450046, China; shjykyzx@163.com (X.L.); 80525@hnuahe.edu.cn (C.P.); 3 College of Science, Henan University of Technology, Zhengzhou 450001, China; junfei_w@126.com; 4 Key Laboratory of Functional Molecules for Biomedical Research, Henan University of Technology, Zhengzhou 450001, China

**Keywords:** NXDQ, microbial community, volatile compounds, biomarkers, correlation analysis, metabolic pathway and differential enzymes

## Abstract

*Nongxiangxing-daqu* (NXDQ), as a saccharification and fermentation agent, directly affects the flavor and yield of fresh *Nongxiangxing Baijiu* (NXBJ). The difference in fermentation temperature owing to the artificial turning operation leads to the formation of superior (S) and normal (N) grades of NXDQ. Here, aiming to explore the discriminant characteristics of two grades of NXDQ, we studied the physicochemical properties, volatile compounds and microbial communities using HS-SPME-GC/MS and high-throughput sequencing technology. The NXDQ grades presented different physicochemical properties. *Staphylococcus*, *Weissella*, *Lactobacillus* and *Thermoascus* were dominant in the S grade (S-NXDQ), while *Bacillus*, *Thermoactinomyces* and *Aspergillus* were predominant in the N grade (N-NXDQ). Higher alcohols, aldehydes and ketones positively correlated with the bacterial biomarkers could be used as metabolic biomarkers for N-NXDQ; the S-NXDQ had a higher abundance of key enzymes involved in lactic acid and ethanol fermentation, while N-NXDQ had a higher abundance of key enzymes involved in amino acid synthesis and long-chain fatty acid and lipid metabolism. N-NXDQ and S-NXDQ had different microbial and metabolic biomarkers. These findings provide insight into the discriminant characteristics of different grades of NXDQ, a theoretical basis for rational evaluation of NXDQ, and effective information for quality improvement of *daqu*.

## 1. Introduction

Chinese *Baijiu* is one of the six distillates in the world [1] and has twelve flavor types. Among them, *Nongxiangxing Baijiu* (NXBJ) has a high output and consumer market [2]. *Daqu*, as a fermentative aroma agent and one of the raw materials for Chinese *Baijiu* brewing, plays a decisive role in the classification of *Baijiu* flavor types and directly affects the yield and quality of fresh *Baijiu* [3]. Medium-temperature *daqu,* with the highest fermentation temperature of 50~59 °C, is commonly used to produce NXBJ, which is also known as NXDQ [4]. The production process of NXDQ is described in our previous reports [5]. During solid-state fermentation under open conditions, a large number of hydrolases and flavor components are enriched through the metabolism of microorganisms from the surrounding environment. After storage for 3–6 months, the useful microorganisms are retained, and the metabolic components have finished transforming and have reached a balance, giving a mature quality to *daqu* [6]. However, the high-quality or table-quality of *daqu*, given the excellent quality of fresh *Baijiu*, is closely monitored by most manufacturers of *Baijiu.*

*Daqu* quality is affected by many factors [7], among which the fermentation temperature is the primary driver of microbial community changes causing differences in quality [8]. At present, *daqu* fermentation temperature is mainly controlled by an artificial turning operation, such as turning over the *daqu* bricks from upper to bottom or outside to center, and switching doors and windows by the skilled workers [9]. The manual operations induce an uneven fermentation environment, including temperature, humidity, acidity and dissolved oxygen concentration, which drive microbial community succession in *daqu* fermentation [8,10] and spatial distribution in *daqu* bricks [11]. The differential microbial communities and distribution affect their metabolic properties, resulting in different qualities of *daqu* bricks in the same production batch [9,12]. To improve or stabilize the quality of *daqu*, it is necessary to comprehensively reveal the essential distinction of different quality grades *of daqu*.

*Daqu* quality is divided into S and N grades or different colors according to appearance, fracture surface and flavor by sensory evaluation [13,14]. However, with the development of omics biotechnology, studies have reported significant differences in microbial composition, volatile metabolites and enzyme activities among the different grades [6,13] or types of *daqu* [15,16]. Comparative analysis on sauce-flavor *daqu* with two grades demonstrated that the dominant genera in S grade were *Lentibacillus*, *Burkholderia*, *Saccharopolyspora*, *Thermoascus* and *Rasamsonia*, while *Staphylococcus* and *Scopulibacillus* were the dominant genera in N grade. In addition, compared to the N grade, the S grade of *daqu* had higher content of differential metabolites, including acids, amino acids and alcohols [13]. Roasted-sesame-like flavored *daqu* yields three grades: premium-, first- and general. The two former have higher liquefication and saccharification activities and more similar microbial genus abundances than the later, while general grade of *daqu* had higher esterification activity. However, the findings indicated that the concentrations and proportions of various esters had no rule linking them to the flavor compound types, and neither biochemical activity nor appearance evaluation would reflect the composition and viability of the microorganisms and enzymes [6]. Yellow *daqu* showed the highest fermenting power, while black *daqu* was opposite but had the highest free amino acid content among three colors (white, yellow and black) of sauce-flavor *daqu* [15,17]. Similarly, three kinds of light-flavor *daqu* (*Qingcha*, *Hongxin* and *Houhuo*) had different predominant microbial communities, physicochemical indices, enzyme activities and volatile flavor compounds [14]. The above studies prove that the same production batch could produce different qualities of *daqu* and each has its own characteristics.

The S and N grades of NXDQ are also formed owing to temperature controls by artificial operation. The S grades not only matche the national light industry standards in physiochemical indices (QB/T 4259-2011) [18], but also excel at sensory characteristics, including uniform white mycelium on the surface, a neat fracture section, a thin raw starch layer and the typical flavor of NXDQ. The N grade does not have the above characteristics. However, current studies on quality differences of *daqu* mainly focus on sauce-flavor and light-flavor, while that of NXDQ is seldom considered. In this study, differential characteristics of the metabolic profiles and microbial community as well as its function between S and N grades of NXDQ based on sensory evaluation were studied using modern high-throughput sequencing technology and HS-SPME-GC/MS, combining multivariate statistical methods. In addition, the potential functions of microbial communities were predicted. This study provides a useful theoretical basis for the scientific evaluation and rational application for the S and N grades of NXDQ, and also provides effective information for quality improvement of *daqu*.

## 2. Materials and Methods

### 2.1. Daqu Quality Evaluating and Sampling

The mature NXDQ with S grade (marked MSH_S and MYP_S) and N grade (marked MSH_N and MYP_N) were collected from two well-known distilleries in Henan province in China. The quality grade was evaluated by experienced *daqu*-making workers and well-trained sensory evaluation experts according to the appearance, the thickness of raw starch layer, fracture section and aroma. For each grade of *daqu*, 10 bricks were randomly selected from the matured *daqu* piles, then were homogenized and mixed into one experimental sample. Each sample was divided into two parts and stored in a sterile bag at either −20 °C for microbial counts, physicochemical properties and volatile compound analysis, or at −80 °C for genomic sequencing analysis.

### 2.2. Culture-Dependent Microbial Counts

The culture-dependent microbial communities in NXDQ samples included aerobic bacteria, lactic acid bacteria and fungi, and were enumerated using the traditional dilution counting method [5]. PCA medium (Oxoid Ltd., Basingstoke, UK), MRS agar medium (Oxoid Ltd., Basingstoke, UK) and PDA agar medium (Oxoid Ltd., Basingstoke, UK) were used to culture for aerobic bacteria, lactic acid bacteria and fungi growth, respectively. Colony forming units (CFU) were calculated as the total number of colonies per gram of fresh weight sample (CFU/g).

### 2.3. Determination of Physiochemical Properties 

Physicochemical properties of *daqu* samples, including moisture, total titration acidity, liquefying power, saccharification power, esterification power and fermentation power, were determined according to the industrial general standard methods (QB/T 4257-2011) [18] and our published methods [5]. Moisture was determined by measuring weight loss after 4~5 g *daqu* samples were dried at 105 °C until constant weight. The total titration acidity (abbreviated as acidity) was determined by calculating the consumed standard 0.1 mol/L NaOH solution to titrate the sample solution with an endpoint at pH 8.2. The liquefying power was determined by measuring the minutes of blue reaction disappearance with iodine and starch solution, and was defined as the mass of the liquefied starch by 1 g *daqu* at 35 °C and pH 4.6 in 1 h. The saccharification power was determined by measuring the consumed standard 0.2% (g/g) glucose solution to titrate the Fehling’s reagent solution containing the saccharification solution of the *daqu* sample, and was defined as the mass of glucose saccharified from starch by 1 g daqu at 35 °C and pH 4.6 in 1 h [19]. Esterifying power was determined using the saponification method and was defined as the mass of total esters synthesized by caproic acid and ethanol at 35 °C for 100 h with 1 g daqu samples. Ferment power is measured by the total carbon dioxide produced in the fermentation of the saccharified sorghum solution inoculated with daqu samples, and was defined as the mass of carbon dioxide fermented from 7°Bé sorghum solution by 0.5 g *daqu* at 30 °C for 72 h. 

### 2.4. Analysis of Volatile Compounds 

The volatile compounds from NXDQ samples were analyzed by HS-SPME-GC/MS. The preconditioned SPME head with a 50/30 μm DVB/CAR/PDMS fiber (Supelco, Bellefonte, PA, USA) was performed to extract the volatile compounds at 50 °C for 45 min. Then, the SPME fiber immediately inserted into the injection port of GC and desorbed at 250 °C for 5 min. The volatile compounds were detected by GC–MS (Trace 1300—TSQ 9000, Thermo Scientific, Waltham, MA, USA) equipped with a trace TR-WAX fused silica capillary column (30 m × 0.25 mm i.d., 0.25 μm film thickness, Thermo Scientific, Waltham, MA, USA). The GC temperature program and mass spectrometry conditions were operated, and volatile compounds were identified using our previously reported methods [5]. The volatile compounds were semi-quantitatively analyzed according to the published methods [7]. The differential volatile compounds were analyzed using the partial least squares discriminant analysis (PLS-DA) by SIMCA (14.1) software, and VIP value (VIP ≥ 1) was performed to select the flavor markers of NXDQ [20].

### 2.5. DNA Extraction and Amplicon High-Throughput Sequencing

Total genomic DNA of NXDQ was extracted using the NucleoSpin Soil Kit (Macherey-Nagel, Düren, Germany) based on the manufacturer’s instructions. The quality and quantity of the extracted DNA was determined by 1% agarose gel electrophoresis and a Qubit Fluorometer by Qubit dsDNA BR Assay kit (Invitrogen, Carlsbad, CA, USA), respectively. PCR amplification of the bacterial 16S rRNA V4 region and the fungal ITS1 region was performed with the universal primer pairs 515F/806R and ITS1F/ITS2R. The primers, master mix and 30 ng template comprised the 50 μL PCR reaction. PCR cycling conditions were as follows: 94 °C for 3 min, 30 cycles of 94 °C for 30 s, 56 °C for 45 s for 16S rRNA and 55 °C for 45 s for ITS1, 72 °C for 45 s and final extension for 10 min at 72 °C. The PCR products were purified and eluted to construct libraries and the validated libraries were ultimately sequenced on the Illumina HiSeq 2500 platform (BGI, Shenzhen, China) generating 2 × 250 bp paired-end reads. Raw reads were filtered to remove adaptors, low-quality and ambiguous bases, and then the paired-end reads were merged by FLASH (v1.2.11, https://ccb.jhu.edu/software/FLASH/index.shtml, accessed on 18 February 2024) [21] to obtain high-quality sequences. The sequencing data were deposited in the NCBI Sequence Read Archive (SRA) database and are available under accession number PRJNA815814.

### 2.6. Bioinformatics and Statistical Analysis

The sequence data were processed by Quantitative Insights Into Microbial Ecology (QIIME v1.9.1, http://qiime.org/install/index.html, accessed on 18 February 2024). The OTUs were clustered from the high-quality sequences with a cut-off value of 97% using UPARSE (v7.0.1090, http://drive5.com/uparse/, accessed on 18 February 2024). After removing chimera, OTU representative sequences were taxonomically classified using RDP Classifier (v2.13, https://sourceforge.net/projects/rdp-classifier/, accessed on 18 February 2024) against the Silva Database (Silva v138, http://www.arb-silva.de/, accessed on 18 February 2024) for bacteria and against the Unite Database (Unite v8.0, http://unite.ut.ee/index.php) for fungi with a minimum confidence threshold of 0.7. USEARCH (v11, http://www.drive5.com/usearch, accessed on 18 February 2024) was used to obtain the OTU abundance statistics table of each sample by comparing all high-quality sequences back to OTUs.

Alpha and beta diversity were evaluated using Mothur (v1.30.2, https://mothur.org/wiki/calculators/, accessed on 18 February 2024) [22] and QIIME (v1.9.1) at the OTU level, respectively, and principal coordinate analysis (PCoA) was used to visualize the beta diversity based on Bray–Curtis dissimilarity. The LDA effect size (LEfSe) method (LDA > 3.5, *p* < 0.05) [23] was performed to estimate the significant variations in microbial genus from different samples, and Wilcoxon rank-sum test was used to investigate the differential genera of two grades of NXDQ. Pathway analysis and key enzyme analysis were performed and the functional composition of microbial community was predicted by PICRUSt2 according to MetaCyc (https://metacyc.org/, accessed on 18 February 2024) and KEGG databases (https://www.kegg.jp/, accessed on 18 February 2024) [24].

Microbial counts, physicochemical properties and volatile compounds among different grades of NXDQ samples were compared using SPSS (version 16.0) by one-way ANOVA and *p* < 0.05 was considered significant. Redundancy analysis (RDA) by R software (version 3.3.1) with vegan packages was used to reveal the correlation between dominant microbial genera and physicochemical properties based on Pearson’s rank [25], and Cytoscape (v3.7.1) was performed to visualize the correlation network between dominant volatile compounds and microbial genera based on Spearman’s rank. The correlation indices |ρ| > 0.6 and *p* < 0.05 were defined as significant relationships. Heatmaps of the key enzymes were drawn by R software (version 3.3.1) with heatmap packages.

## 3. Results

### 3.1. Cultivable Microbial Counts of NXDQ Samples

**Figure 1:**
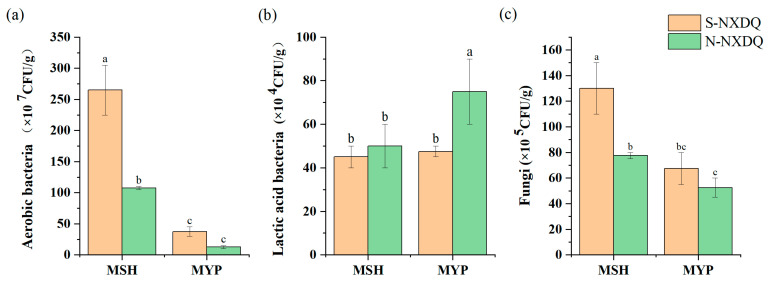
Cultivable microbial counts in S-NXDQ and N-NXDQ. (**a**) Cultivable aerobic bacterial counts. (**b**) Lactic acid bacterial counts. (**c**) Fungal counts. Letters above bars indicate significance markers, and different letters were significantly different (*p* < 0.05).

The counts of cultivable microorganisms in two grades of NXDQ are shown in Figure 1. The total aerobic bacteria and fungi were significantly higher in MSH_S than in MSH_N. There was no significant difference in the distribution of aerobic bacteria and fungi in MYP_S and MYP_N. The lactic acid bacterial counts in the two grades of MSH were not significantly different, while that of MYP_N was significantly higher than that of MYP_S.

### 3.2. Comparison of Physicochemical Properties

Table 1 shows the differences in physicochemical properties between S- and N-NXDQ. It can be seen that moisture in NXDQ ranged from 11.30% to 13.33%, which is less than the 15% maximum value of moisture in daqu regulated by the national standards (QB/T 4259-2011, 2011) [18]. However, the moisture in S-NXDQ (11.30~11.80%) was significantly lower than that of N-NXDQ (12.27%~13.33%). The acidity ranged from 0.58 to 2.15 mmol/10 g, and the acidity of MSH was significantly lower than that of MYP, while the acidity in MYP_N was significantly higher than that of MYP_S (*p* < 0.05). Additionally, MYP_S had higher liquefaction power and saccharification power, and MSH_S had higher ferment power, while MYP_N had the higher esterification power.

### 3.3. Microbial Community α-Diversity and β-Diversity between S- and N-NXDQ

**Figure 2:**
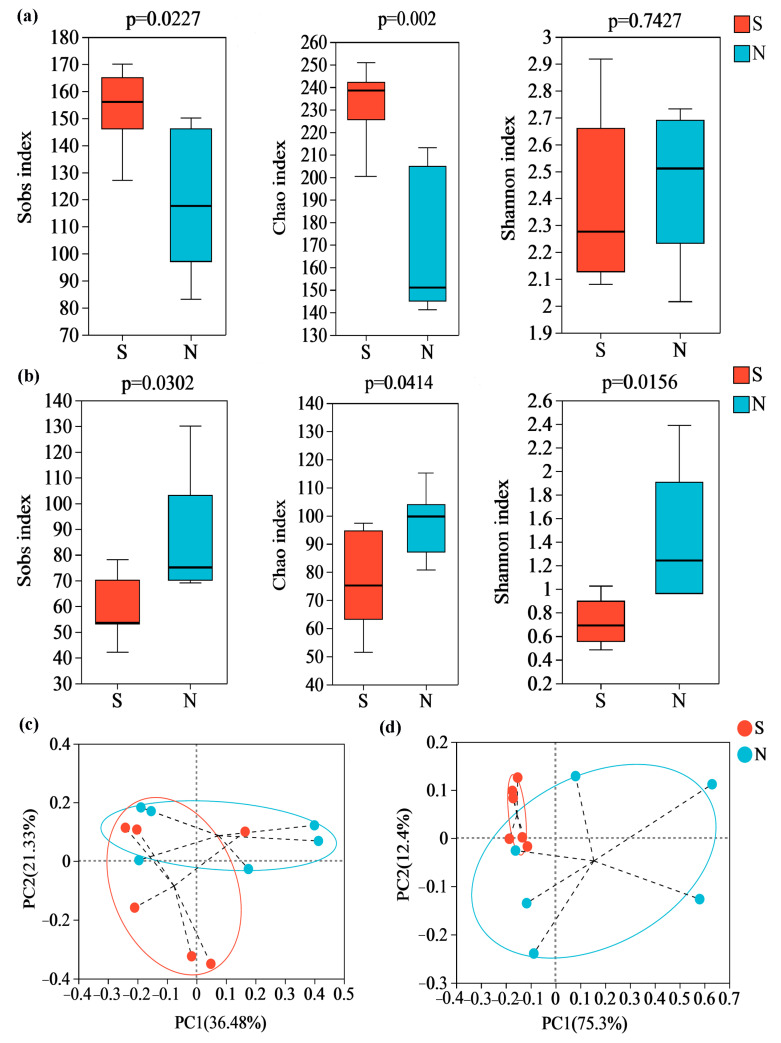
α-diversity and β-diversity analysis of the microbial community in two grades of NXDQ. (**a**) α-diversity indexes for bacterial community. (**b**) α-diversity indexes for fungal community. (**c**) PCoA analysis for bacterial community. (**d**) PCoA analysis for fungal community.

An average of 24,227 bacterial valid sequences and 69,960 fungal valid sequences were found per *daqu* sample after quality filtering, denoising, removing chimeras and sequence leveling. Then, along with 350 and 522 fungal OUTs for S and N-NXDQ, 920 and 711 bacterial OUTs were obtained, respectively. The Sobs, Chao and Shannon index, as α-diversity indices, reflected the observed species, community richness and community diversity, respectively. As shown in Figure 2, the bacterial Sobs and Chao indexes of S-NXDQ were significantly higher than that of N-NXDQ (Figure 2a), while two indexes of fungi were the opposite (Figure 2b). The bacterial Shannon index was not significantly different between the two grades, while the fungal Shannon index of N-NXDQ was significantly higher than that of S-NXDQ. The results indicated that the N-NXDQ *daqu* had a richer and more diverse fungal community, while the bacterial community in S-NXDQ was richer. The PCoA showed that there was a significant separation between S- and N-NXDQ (Figure 2c), indicating that the microbial structure in the two grades of NXDQ had different characteristics.

### 3.4. Microbial Community Composition and Difference between S- and N-NXDQ

**Figure 3:**
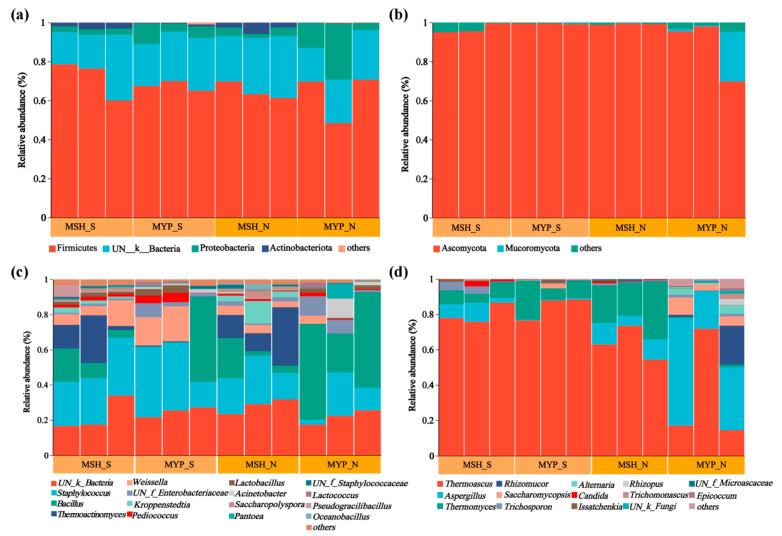
Relative abundance of the microbial communities in S-NXDQ and N-NXDQ samples. (**a**) Bar plots of bacterial communities at the phylum level. (**b**) Bar plots of fungal communities at the phylum level. (**c**) Bar plots of bacteria at the genus level. (**d**) Bar plots of fungi at the genus level.

The relative abundance (more than 1%) of microbiota composition at phylum and genus level is shown in Figure 3. Four bacterial phyla, including Firmicutes, unclassified_K_Bacteria (UN_K_Bacteria), Proteobacteria and Actinobacteria (Figure 3a), and two fungal phyla, including Ascomycota and Mucoromycota (Figure 3b), were found to be the dominant phyla, which accounted for more than 98% of the total microorganisms. There was no obvious difference in the composition of bacterial and fungal phyla between S- and N-NXDQ, while the average relative abundance of the dominant three fungal phyla in MYP_N was higher than that in MYP_S, respectively. Sixteen bacterial genera and fourteen fungal genera were found. The dominant bacterial genera were UN_k_*Bacteria*, *Staphylococcus*, *Bacillus*, *Thermoactinomyces*, *Weissella*, UN_f_*Enterobacteriaceae* and *Kroppenstedtia* (Figure 3c). Furthermore, the average abundance of *Staphylococcus* (29.65%) and *Weissella* (10.33%) in S-NXDQ was obviously higher than that (17.28% and 2.98%) in N-NXDQ, while the average abundance of *Bacillus* (26.75%) and *Thermoactinomyces* (9.44%) in N-NXDQ was higher than that (13.58% and 7.24%) in S-NXDQ. The fungal genera was dominated by *Thermoascus*, *Aspergillus*, *Thermomyces*, *Rhizomucor* and *Saccharomycopsis* (Figure 3d). Meanwhile, *Thermoascus* was relatively higher in S-NXDQ (82.09%) than that in N-NXDQ (48.96%). On the contrary, *Aspergillus* presented the highest average abundance in N-NXDQ (24.52%), especially in MYP_N (39.26%), which was obviously higher than that in S-NXDQ (3.84%). *Thermoascus* and *Aspergillus* accounted for more than 74% of the fungal community in N-NXDQ. In addition, the average abundance of *Rhizomucor* and *Saccharomycopsis* in N-NXDQ was higher than that in S-NXDQ.

**Figure 4:**
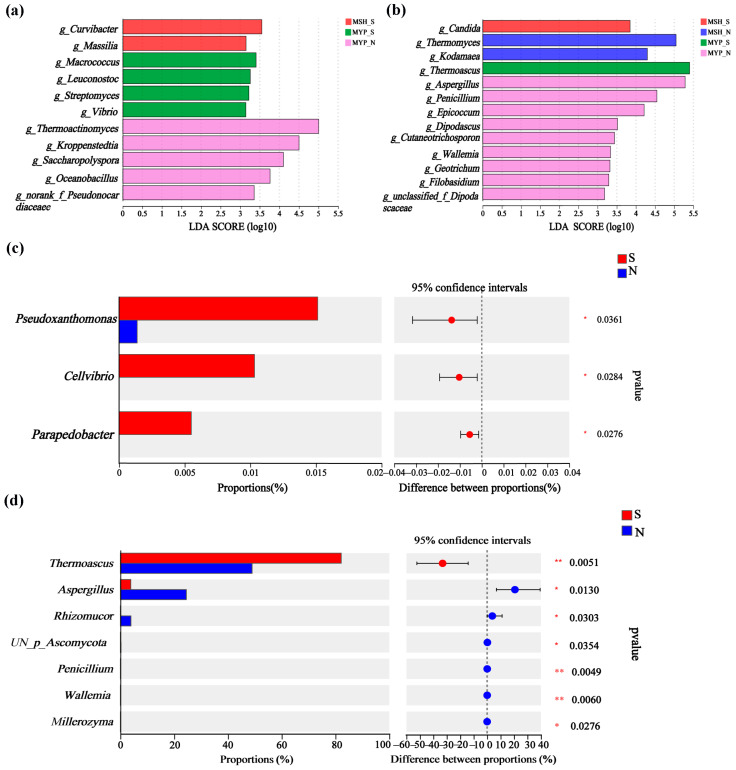
The variations of the microbial communities in two grades of NXDQ based on linear discriminant analysis (LDA) effect size (LEfSe) and Wilcoxon rank-sum test. (**a**) The LDA score indicated the discriminant level of bacterial genera. (**b**) The LDA score indicated the discriminant level of fungal genera. (**c**) Comparison of variance bacterial genera. (**d**) Comparison of variance fungal genera. LDA > 3.5, *p* < 0.05, the levels of significance: * 0.01 < *p* ≤ 0.05; ** *p* ≤ 0.01.

Based on the linear discriminant analysis (LDA) effect size (LEfSe) and Wilcoxon rank-sum test, the variance in microorganisms in the two grades of NXDQ are shown in Figure 4. Eleven bacterial genera and thirteen fungal genera were identified as differential taxa with significantly different relative abundance (*p* < 0.05). MSH_N and MYP_N had more differential bacteria and fungi, respectively. *Curvibacter*, *Macrococcus* and *Thermoactinomyces* were significantly enriched in MYP_S, MSH_S and MSH_N samples, respectively. Meanwhile, *Candida*, *Thermomyces*, *Thermoascus* and *Aspergillus* were enriched in MSH_S, MSH_N, MYP_S and MYP_N samples, respectively. The rank-sum test showed that *Pseudoxanthomonas*, *Cellvibrio* and *Parapedobacter* in S-NXDQ were significantly higher than those in N-NXDQ. In the fungal genera, except for *Thermoascus*, the relative abundance of the other six fungal genera in N-NXDQ was significantly higher than that in S-NXDQ. These differential bacteria and fungi could be used as biomarkers.

### 3.5. Volatile Compounds Composition

**Figure 5:**
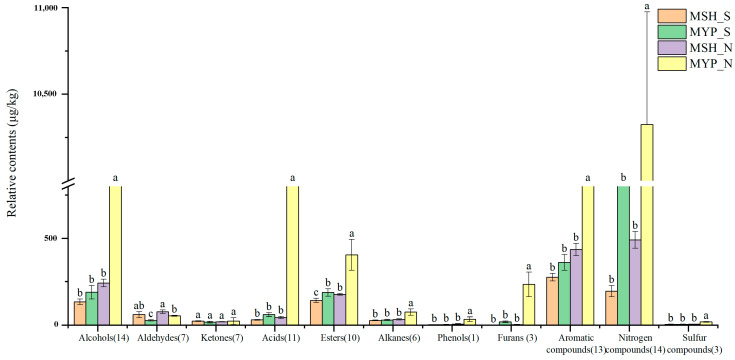
Relative content of the dominant categories of volatile compounds in two grades of NXDQ. Different letters represent significant differences (*p* < 0.05).

**Figure 6:**
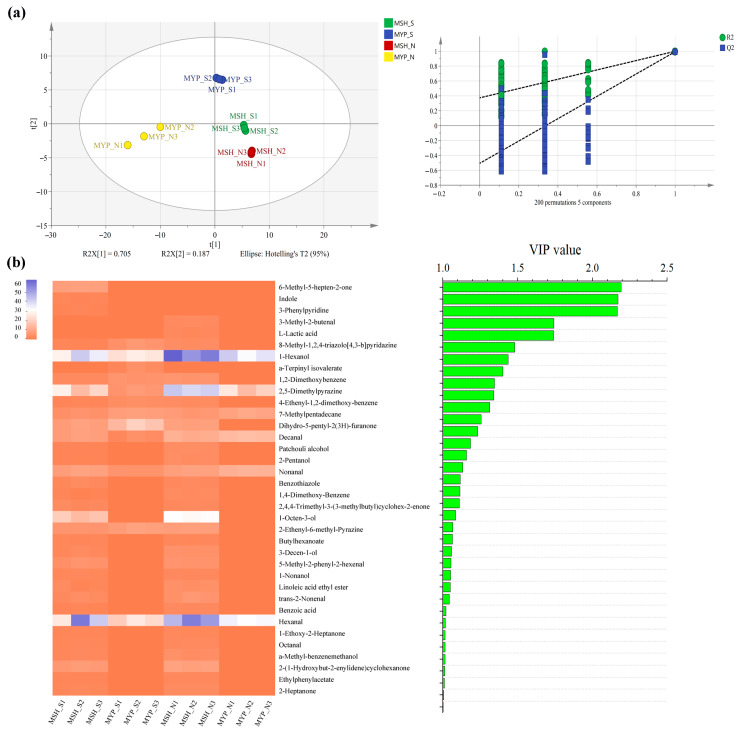
Partial least squares discriminant analysis (PLS-DA) of the volatile compounds (**a**) and heatmap and bar chart of the differential volatile compounds between two grades of NXDQ selected by PLS-DA (**b**) (VIP score >1.0, *p* < 0.05).

A total of ninety-seven volatile compounds, including alcohols, aldehydes, ketones, acids, esters, alkanes, phenols, furans, aromatic compounds, nitrogen-containing compounds, sulfur-containing compounds and others, was determined (Supplementary Materials Table S1). Figure 5 indicated that the content of most of the volatile compounds was not significantly different between MSH_S and MSH_N, while the content of dominant volatile compounds in MYP_N was significantly higher than that in other samples. The results of the OPLS-DA model based on volatile compounds (Figure 6a) showed that S- and N-NXDQ were significantly separated. Meanwhile, the R^2^Ycum and Q^2^cum values were 0.966 and 0.944, respectively, suggesting that the model had good accuracy and predictability. Thirty-six differential volatile compounds, including six alcohols, eleven aldehydes and ketones, four esters, six aromatic compounds, four nitrogen-containing compounds and five other compounds, were identified by the VIP analysis (Figure 6b). The total of alcohols, aldehydes and ketones was seventeen, which accounted for approximately half of the total differential compounds, and had the higher content in N-NXDQ. Except for 2,5-Dimethylpyrazine, the content of other nitrogen-containing compounds in S-NXDQ was higher than that in N-NXDQ (Figure 6b). These compounds could be used as biomarkers of volatile metabolites.

### 3.6. Dominant Microbial Relationships with the Physicochemical Properties and the Differential Volatile Compounds

**Figure 7:**
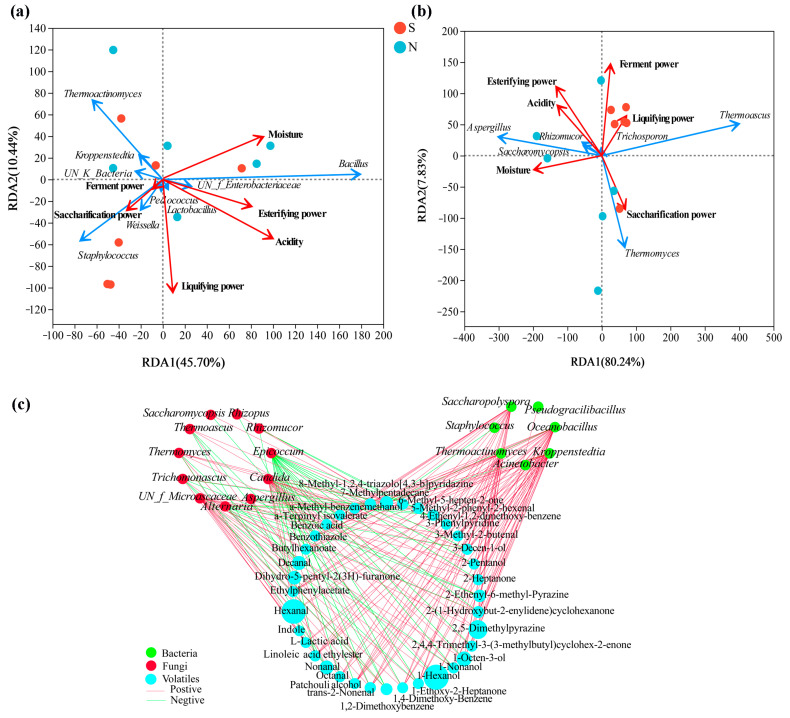
RDA of the relationship between physicochemical properties and (**a**) bacterial genera and (**b**) fungal genera, and the network visualization of the correlation (**c**) between microbial genera and discriminatory volatile compounds. The red arrows represent physicochemical indexes, the blue arrows represent bacterial and fungal genera, and the arrow length represents the degree of relationship. The nodes represent the number of microbial genera and volatile compounds (VIP score > 1.0) which are significantly related, the red edge and the green edge represent the positive correlation (ρ > 0.7 and *p* < 0.05) and the negative correlation (|ρ| > 0.7, ρ < 0, and *p* < 0.05), respectively.

RDA based on Pearson’s rank correlation was used to evaluate the correlation between the top ten bacterial or top ten fungal genera and the physicochemical properties. The two axes explained 56.14% and 88.07% of the total variance in bacteria (Figure 7a) and fungi (Figure 7b) community differentiation, respectively, suggesting remarkable correlations. Figure 7a shows that S-NXDQ was closely related to saccharification power, liquifying power and ferment power, while N-NXDQ was relatively more dispersed. However, MYP_N was related to acidity, moisture and esterifying power. Moreover, *Staphylococcus* and *Weissella* were positively correlated with saccharifying power, liquefying power and ferment power. Acidity, moisture and esterification power were positively correlated with *Bacillus*, UN_f_*Enterbacteriaceae* and *Lactobacillus*, while negatively correlated with *Staphylococcus*, *Weissella*, UN_k_*Bacteria*, *Kroppenstedtia* and *Thermoactinomyces*. Figure 7b shows that S-NXDQ was positively correlated with liquifying power and ferment power, while N-NXDQ was positively correlated with moisture. Moreover, *Thermoascus* and liquefying power were positively correlated, as were *Thermomyces* and saccharification power. Meanwhile, *Aspergillus*, *Saccharomycopsis* and *Rhizomucor* were positively correlated with moisture, acidity and esterifying power.

The correlations between dominant microorganisms (the top twenty bacterial genera and top twenty fungal genera) and thirty-six differential volatile compounds were evaluated by Spearman’s rank correlation analysis (Spearman’s correlation test, |ρ| > 0.6, *p* < 0.05), and were visualized by network (Figure 7c). As seen from correlation network, seven bacteria were positively correlated with most of the thirty-six volatile compounds, while eleven fungi were negatively correlated with more volatile compounds. Furthermore, most of the alcohols, aldehydes and ketones were strong positively associated with *Thermoactinomyces*, *Kroppenstedtia*, *Saccharopolyspora*, *Oceanobacillus* and *Thermomyces*, and most of the aromatic compounds and nitrogen-containing compounds were positively correlated with *Thermoascus* while negatively correlated with *Aspergillus*, indicating that the differential metabolic compounds in two grades of NXDQ were closely related to the characteristic microorganisms.

### 3.7. Potential Function Prediction and Metabolic Pathways of the Microbial Communities in S- and N-NXDQ

**Figure 8:**
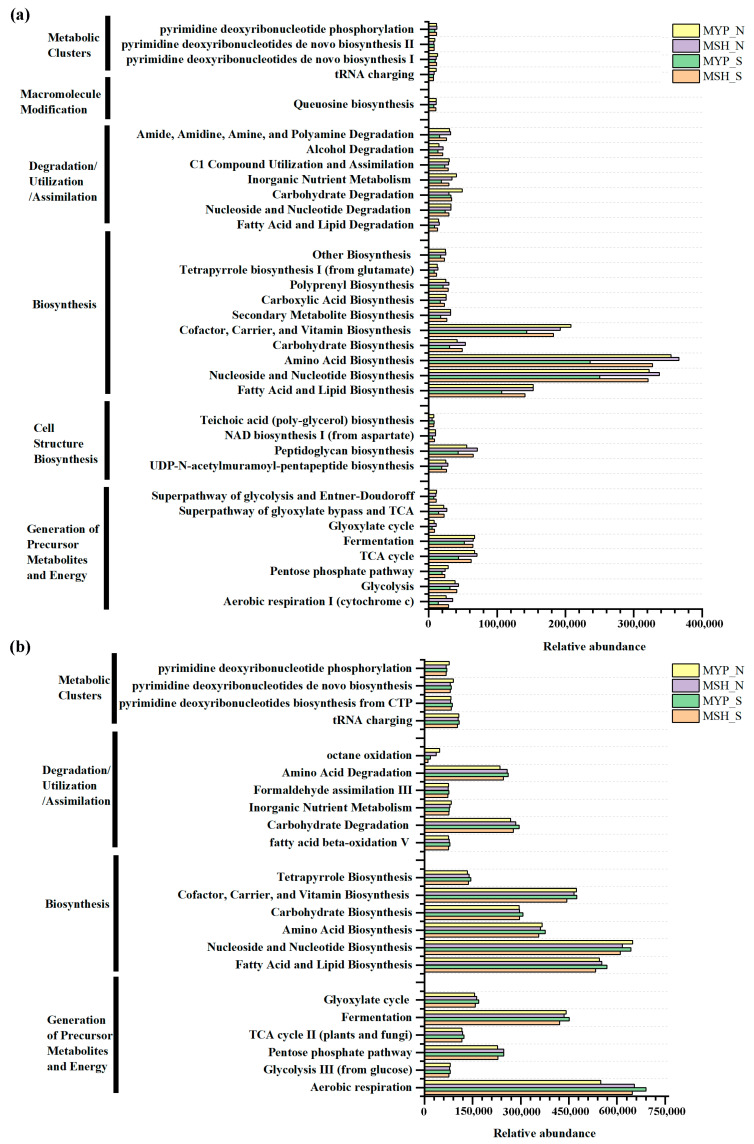
Abundance of microbial metabolic pathways in two grades of NXDQ. (**a**) Bacterial metabolic pathways. (**b**) Fungal metabolic pathways.

Based on commercial databases of KEGG and MetaCyc [26], the metabolic pathway enrichment was analyzed. A total of three hundred and ninety-nine bacterial metabolic pathways and seventy-four fungal metabolic pathways were obtained in the context of MetaCyc analysis. It can be observed from Figure 8a, that bacterial communities mainly presented six metabolic pathways, including metabolic clusters, macromolecule modification, degradation/utilization/assimilation, biosynthesis, cell structure biosynthesis, and generation of precursor metabolites and energy, while fungal community mainly detected four pathways, including metabolic clusters, degradation/utilization/assimilation, biosynthesis, and generation of precursor metabolites and energy (Figure 8b). Furthermore, the metabolic pathways in two grades of NXDQ mainly focused on the bacterial biosynthesis and on the fungal biosynthesis and generation of precursor metabolites and energy. Compared with the bacterial community, the fungal community had a higher abundance of dominant metabolic pathways, especially in carbohydrate degradation, amino acid degradation, fatty acid and lipid biosynthesis and fermentation. However, the differences of the bacterial metabolic pathways between S- and N-NXDQs were more obvious.

**Figure 9:**
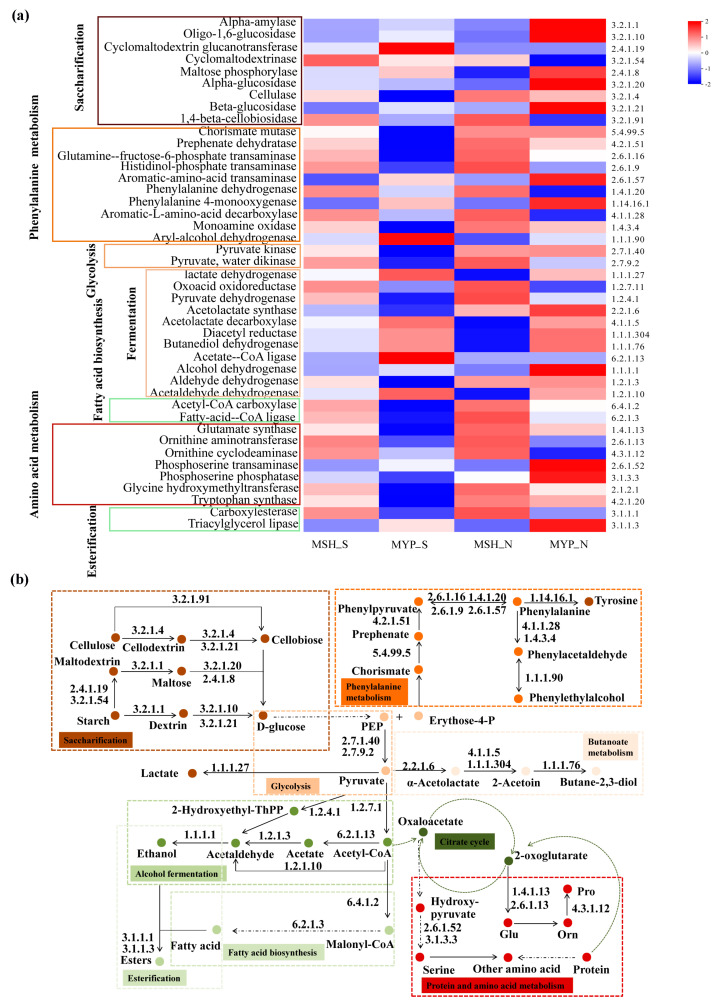
Potential functions of the enzyme-encoded bacterial community in different grades of NXDQ. (**a**) Profiles of the key enzymes related to the dominant metabolic pathway based on 16S rRNA gene sequences. (**b**) Predominant metabolic pathway based on dominant enzyme-encoded bacterial community.

According to the volatile compounds taxa, the metabolic pathways in NXDQ were classified into nine categories: starch saccharification, glycolysis, phenylalanine metabolism, butanoate metabolism, alcohol fermentation, fatty acid biosynthesis, esterification, TCA cycle, protein and amino acid metabolism (Figure 9b). To further reveal the difference of metabolic pathway enrichment, the enzymes metabolized by bacterial communities were carried out through KEGG analysis. A total of two thousand and eighty-three enzymes were detected, and the forty-three enzymes that played a key role in metabolic pathways were selected to analyze their difference between S- and N-NXDQ. As shown in Figure 9a, the abundance of enzymes in S-NXDQ, including Cyclomaltodextrin glucanotransferase (EC2.4.1.19), Cyclomaltodextrinase (EC3.2.1.54), Aryl-alcohol dehydrogenase (EC1.1.1.90), Lactate dehydrogenase (EC1.1.1.27), Acetolactate decarboxylase (EC4.1.1.5), Acetate-CoA ligase (EC6.2.1.13) and Acetaldehyde dehydrogenase (EC1.2.1.10), were higher than that in N-NXDQ. These enzymes were mainly involved in the first step of starch degradation, conversion of phenylethyl alcohol and phenylacetaldehyde, lactic acid and acetoin synthesis, acetyl-CoA and acetaldehyde synthesis. However, the abundances of 16 enzymes in N-NXDQ were higher than that in S-NXDQ. Sixteen enzymes mainly included some amylases, phenylalanine and phenylacetaldehyde forming enzymes, pyruvate formation and degradation enzymes, long-chain fatty acid synthase, tryptophan and other amino acid synthase.

## 4. Discussion

The physicochemical properties of *daqu* were related to the microbiota composition. N-NXDQ had the higher moisture and acidity, and could provide relatively suitable humidity and acidity for fungal growth and survival during *daqu* aging. Therefore, compared with *Thermoascus* dominating in S-NXDQ, N-NXDQ had more predominant fungal taxa, which included *Thermoascus*, *Aspergillus*, *Thermomyces*, *Rhizomucor*, *Saccharomycopsis* and *Alternaria*. The previous findings showed that the humidity was negatively related to *Thermoascus*, while the acidity was positively related to *Aspergillus* and *Rhizomucor* [27], indicating that the suitable moisture and acidity were conducive to the growth of more fungal taxa. However, the S-NXDQ was dominated by *Thermoascus* and *Thermomyces*. The reason could be that in high-temperature fermentation of S-NXDQ, temperature eliminated most of the molds and yeasts that were not resistant to the high temperature, while enriching the *Thermoascus* and *Thermomyces* that are tolerant to high-temperature [28]. *Thermoascus* and *Thermomyces* could metabolize liquefaction enzyme, glucoamylase [16], thermophilic glycoside hydrolase, and could also participate in the degradation of lignocellulose [28], which is consistent with our findings. We found a higher relative abundance of *Thermoascus*, higher liquefaction power and saccharification power in S-NXDQ. Similarly, a strong positive correlation between *Thermoascus* and liquefaction power, and between *Thermomyces* and saccharification power, was indicated by RDA. These results again confirmed that heat-resistant filamentous fungi contribute to the degradation of macromolecular starch in S-NXDQ. Inversely, some fungal genera, such as *Rhizomucor*, could secrete lactic acid and contribute to the acidity formation [29]. This could be one of the reasons that a higher acidity was detected in MYP_N.

The dominant bacterial composition between S- and N-NXDQ was also an observable difference. The average relative abundances of *Staphylococcus*, *Lactobacillus* and *Weissella* were higher in S-NXDQ than that in N-NXDQ, while the average relative abundances of *Bacillus* and *Thermoactinomyces* in N-NXDQ were significantly higher than that in S-NXDQ. This was opposite to the bacterial genera composition of the high-temperature *daqu* with superior and normal grades [16]. *Lactobacillus* and *Weissella*, belonging to lactic acid bacteria, were the dominant genera in mature medium-temperature *daqu* [30] and contribute to the formation of acidity and flavor compounds [31,32], which is consistent with our findings. In contrast, *Bacillus* was dominant in N-NXDQ, while the abundances of *Lactobacillus* and *Weissella* decreased, which could be explained by the fact that *Bacillus* inhibited the growth of lactic acid bacteria [33]. In addition, the enriched *Bacillus* could be related to the locally high fermentation temperature in N-NXDQ.

The difference of microbial communities in two grades of NXDQ induced the differences of metabolites. Among eleven kinds of volatile compounds, the total content of nitrogen-containing compounds was the highest, followed by esters and alcohols. Pyrazines, as nitrogen-containing compounds with baking and roasting aromatic [34], were identified as the main volatile compounds in sauce-flavor *daqu* [13]. However, the nitrogen-containing compounds dominated by tetramethylpyrazine had the highest content in N-NXDQ, which was related to the metabolism of amino acids and the higher relative abundance of *Bacillus*. This was consistent with the results of the relatively higher abundance of amino acid synthesis pathways in N-NXDQ and *Bacillus* could be helpful to increase the content of tetramethylpyrazine in *daqu*. Ethylhexanoate which presents the main flavor of NXBJ is dominant in esters of NXDQ, and is beneficial to improve the quality of fresh NXBJ [32]. However, we found that N-NXDQ had the higher content of esters, which could be related to the higher content of acids, alcohols or their precursors, and higher esterification power, and could also be related to the composition of fungi. It is worth noting that alcohols and aldehydes also had higher content in N-NXDQ, which is consistent with the previous findings [6]. Alcohols are formed from the alcohol fermentation pathway by *Saccharomyces cerevisiae*, molds and some bacteria [35], and play an aroma-enhancing role in *Baijiu*. However, too high content of higher alcohols and aldehydes can easily make *Baijiu* give a strong sense of stimulation, and even give a bitter taste. Therefore, N-*daqu* with higher content of higher alcohols and aldehydes could be detrimental to improving the quality of *Baijiu*. Naturally, the metabolic mechanism of the alcohols and aldehydes, and their appropriate ratio with other flavor compounds is worthy of further study. The higher content of most volatile compounds detected in N-NXDQ could be related to the higher abundances of *Bacillus*, *Thermoactinomyces* and *Aspergillus*. However, three taxa were dominant in sauce-flavor *daqu*. *Bacillus* could produce organic acids [36], amino acids [25] and pyrazines [37], and co-culture of *Bacillus licheniformis* with in situ microorganism of *daqu* promote *Aspergillus* to provide more flavor compound [38]. Therefore, *Bacillus* composition and abundance directly affect the quality of *daqu*, and even determine the quality grade of the fresh *Baijiu* [39,40]. *Aspergillus* had a function of high-yield neutral proteases [39]. Under the digestion of proteases, amino acids from protein could be used as one of the substrates for Maillard reaction. The reaction products play an important role in the main flavor compounds of sauce-flavor *Baijiu* [41]. However, it is worth noting that some species of *Aspergillus*, such as *Aspergillus flavus*, can metabolize and secret mycotoxins in grains [42]. Although we have not found the mycotoxins biosynthesis pathway by analysis of metabolic pathways with relatively higher abundance using commercial databases of KEGG and MetaCyc, we suggest that the mycotoxins production and their metabolic activity by *Aspergillus* with higher abundance in N-NXDQ need further study. *Thermoactinomyces* was another functional bacteria that promoted the formation of volatile compounds [9].

Different types of *daqu* have unique microbial community compositions and flavor characteristics. *Bacillus*, *Thermoactinomyces*, *Thermomyces*, *Byssochlamys*, *Trichoderma* and *Aspergillus* were predominant in high-temperature *daqu* [40,43,44], whose metabolites provide the unique flavor characteristics for sauce-flavor *daqu*. However, *Staphylococcus*, *Lactobacillus*, *Weissella*, *Thermoascus*, *Thermomyces and Aspergillus* were predominant in medium-temperature *daqu*, whose metabolites provide the unique flavor characteristics for NXDQ [2,32]. Fortunately, S-NXDQ in our study had the above dominant microbiota composition. NXDQ combined with high-temperature *daqu* is helpful to improve the quality of *Baijiu*.

Amino acids produced by amino acid biosynthesis pathways could participate in the Maillard reaction and contribute to the formation of nitrogen-containing flavors in NXDQ [45]. The abundance of nitrogen-containing volatiles in the N-NXDQ was the highest, indicating that more amino acids were involved in the Maillard reaction under high temperature conditions. The bacterial and fungal communities in two grades of NXDQ had a higher abundance of fermentation pathways. Generally, *daqu* with high ferment power has strong flavor, and the ferment power of high quality *daqu* is higher than that of ordinary *daqu* [6]. In this study, we also found that the ferment power of MSH_S was significantly higher than that of other samples. Meanwhile, the abundances of lactate dehydrogenase (EC1.1.1.27), acetolactate decarboxylase (EC4.1.1.5), acetyl-CoA ligase (EC6.2.1.13) and acetaldehyde dehydrogenase (EC1.2.1.10) which are involved in the lactic acid and ethanol fermentation of bacterial community in S-NXDQ were higher than those in N-NXDQ. However, the abundance of the bacterial fermentation pathway in the sample MSH_S was not significantly different from that of other samples. The reason could be that the pathway was involved in other fermentation pathways in addition to lactic acid and ethanol fermentation. Additionally, the abundances of fatty acid and lipid biosynthesis pathways were also higher in the two grades of NXDQ. Esters of lipids are important components of *daqu* flavor compounds, and fatty acids are one of the substrates for the synthesis of esters. Therefore, it is of great significance for fatty acid and lipid biosynthesis to form flavor compounds of *daqu*. We found that the abundance of bacterial fatty acid and lipid biosynthesis pathways of N-NXDQ was higher than those of S-NXDQ. Long-chain fatty acid synthases, including acetyl-CoA carboxylase (EC6.4.1.2), long-chain fatty acid CoA ligase (EC6.2.1.3) and ester synthase (EC3.1.1.1) were also found to be abundant in N-NXDQ, suggesting that the bacterial communities in N-NXDQ were more active in metabolizing long-chain fatty acids and more conducive to the synthesis of long-chain fatty acid esters, which is consistent with the higher content of esters detected in N-NXDQ. Too high content of long-chain fatty acid ethyl ester has a masking effect on the flavor of *Baijiu*, and can easily produce a greasy taste. The appropriate concentration is beneficial to reduce the nasal irritation and to improve the flexibility of *Baijiu*. Therefore, reasonable control of the concentration of long-chain fatty acid esters in NXDQ is great of significance for *Baijiu* brewing.

## 5. Conclusions

By analyzing the quality properties, microbiota composition and their functions in NXDQ with S and N grades, we determined that their characteristics are different. The N-NXDQ had higher acidity, esterification power, a predominant bacterial and fungi composition that is similar to that in high-temperature *daqu*, and a higher abundance of higher alcohols, aldehydes and ketones, while S-NXDQ had higher liquefaction power, saccharification power and ferment power, and had a typical bacterial and fungal composition of NXDQ. In 36 differential volatile compounds, the alcohols, aldehydes and ketones accounted for approximately 50% of the total, and had higher abundances in N-NXDQ. Furthermore, most of them were positively correlated with the bacterial biomarkers. The metabolic pathways of bacterial community uncovered that S-NXDQ had the higher expression abundance of key enzymes involved in lactic acid and ethanol fermentation, while N-NXDQ had the higher expression abundance of key enzymes involved in amino acid synthesis and long-chain fatty acid and lipid metabolism. Based on the above results, we suggest that the evaluation standard of NXDQ quality should not only focus on sensory indices and biochemical properties, but microbial community position and microbial biomarkers might be a more important contribution for quality of *daqu*. In addition, the rational mixture of S and N grades of *daqu* is also a way to stabilize or improve the quality of fresh *Baijiu*. Meanwhile, our findings also provide effective information for the quality improvement of NXDQ.

## Figures and Tables

**Table 1:** Physicochemical properties of S-NXDQ and N-NXDQ.

NXDQ	Sample	Moisture (%)	Acidity (mmol/10 g)	Liquifying Power (g/g·h)	Saccharification Power (mg/g·h)	Esterifying Power (mg/g·100 h)	Ferment Power (gCO_2_/g·72 h)
S-NXDQ	MSH_S	11.30 ± 0.49 ^c^	0.60 ± 0.07 ^c^	2.34 ± 0.06 ^c^	366.00 ± 50.91 ^c^	27.74 ± 1.73 ^b^	0.49 ± 0.01 ^a^
	MYP_S	11.80 ± 0.07 ^c^	1.70 ± 0.02 ^b^	3.85 ± 0.11 ^a^	519.00 ± 10.61 ^a^	25.71 ± 5.00 ^b^	0.30 ± 0.04 ^b^
N-NXDQ	MSH_N	12.27 ± 0.52 ^b^	0.58 ± 0.03 ^c^	2.26 ± 0.01 ^c^	462.00 ± 72.00 ^ab^	22.56 ± 4.54 ^bc^	0.25 ± 0.08 ^b^
	MYP_N	13.33 ± 0.88 ^a^	2.15 ± 0.56 ^a^	2.65 ± 0.07 ^b^	375.00 ± 72.12 ^c^	33.64 ± 0.31 ^a^	0.33 ± 0.02 ^b^

Values represent means ± SDs (n = 3). Superscript letters after values are significance markers, and different letters were significantly different (*p* < 0.05).

## Data Availability

The original contributions presented in the study are included in the article/Supplementary Material, further inquiries can be directed to the corresponding author.

## Supplementary Materials

The following supporting information can be downloaded at: https://www.mdpi.com/article/10.3390/foods13060914/s1. Table S1: Identification and relative abundance of volatile compounds in the S-NXDQ and N-NXDQ samples.
