# Supplementary material for: Differential Characteristics of the Metabolic Profiles and Microbial Community between Superior and Normal Grades of Nongxiangxing-daqu
Source: Foods. 2024 Mar 17;13(6):914. doi: 10.3390/foods13060914 (PMC10969756; doi:10.3390/foods13060914)
Supplement: Supplementary file 1 [file foods-13-00914-s001.zip › foods-2900935-supplementary.pdf]

Supplemenary Materials

1 Supplementary Tables

Table S1 Identification and relative abundance of volatile compounds in the S-NXDQ and N-NXDQ samples.

Table S1. Identification and relative abundance of volatile compounds in the S-NXDQ and N-NXDQ samples..

**Serial number**	**Retention time (min)**	**CAS Number**	**Volatile compound**	**Contents of volatile compounds (μg/kg)**							
				S-NXDQ samples				N-NXDQ samples			
				**MSH_S**	**SD**	**MYP_S**	**SD**	**MSH_N**	**SD**	**MYP_N**	**SD**
**Alcohols(14)**											
1	4.5	26184-62-3	(S)-(+)-2-Pentanol	1.39	0.15	0	0	2.98	0.44	0	0
2	8.05	123-51-3	3-methyl-1-Butanol	26.42	1.16	79.54	12.67	39.88	2.44	225.96	7.24
3	9.72	71-41-0	1-Pentanol	10.24	1.38	4.65	0.04	15.51	0.19	0	0
4	12.35	556-82-1	Prenol	6.28	1.33	18.33	3.59	21.63	1.06	195.98	66.97
5	13.47	111-27-3	1-Hexanol	30.86	6.22	21.99	1.6	50.77	6.22	32.61	6.07
6	16.8	3391-86-4	1-Octen-3-ol	14.78	1.94	0	0	27	1.09	0	0
7	16.9	112-23-2	1-Heptanol	3.61	0.71	0	0	4.74	0.86	0	0
8	18.02	104-76-7	2-Ethyl-1-Hexanol	5.18	0.05	5.05	0.75	6.15	1.72	0	0
9	19.55	24347-58-8	[S-(R*,R*)]-2,3-Butanediol	7.87	0.47	26.41	8.09	14.31	1.05	785.78	136.29
10	20.69	19132-06-0	2,3-Butanediol	8.26	0.61	12.96	3.87	13.8	0.98	320.87	198
11	21.84	26001-58-1	(Z)-3-Decen-1-ol	2.27	0.43	0	0	4.06	0.32	0	0
12	23.16	124-11-8	1-Nonanol	2.07	0.1	0	0	2.3	0.06	0	0
13	34.47	5986-55-0	Patchouli alcohol	1.45	0.07	0	0	3.22	0.15	0	0
14	35.86	35727-45-8	Ledol	13.24	1.57	19.76	7.9	35.53	5.65	66.56	39.97
**Aldehydes(7)**											
1	2.72	66-25-1	Hexanal	36.95	14.55	19.52	2.93	45.43	6.99	28.78	1.93
2	7.54	107-86-8	3-Methyl-2-butenal	0	0	0	0	2.65	0.11	0	0
3	11.07	124-13-0	Octanal	2.06	0.12	0	0	2.53	0.16	0	0
4	14.77	124-19-6	Nonanal	7.22	0.61	4.6	0.05	7.23	0.65	10.98	0.79
5	18.21	112-31-2	Decanal	6.73	0.62	2.98	0.99	9.83	1.01	13.06	0.61
6	19.28	18829-56-6	trans-2-Nonenal	2.86	0.45	0	0	4.72	1.11	0	0

7	32.68	21834-92-4	5-Methyl-2-phenyl-2-hexenal	4.24	0.59	0	0	4.59	0.24	0	0
**Ketones(7)**											
1	6.88	110-43-0	2-Heptanone	2.07	0.12	0	0	2.85	0.06	0	0
2	10.9	111-13-7	2-Octanone	3.71	0.03	1.98	0.66	4.15	0.05	0	0
3	12.85	110-93-0	6-Methyl-5-hepten-2-one	7.19	0.33	0	0	0	0	0	0
4	15.17	1669-44-9	3-Octen-2-one	2.53	0.59	0	0	3.47	0.53	0	0
5	23.02	51149-70-3	1-Ethoxy-2-Heptanone）	1.7	0.02	0	0	2.1	0.16	0	0
6	27.09	930-30-3	2-Cyclopenten-1-one	0	0	15.46	4.4	0	0	23.1	19.36
7	37.11	132305-28-3	2-(1-Hydroxybut-2-enylidene)cyclohexanone	5.87	0.49	0	0	7.3	0.44	0	0
**Acids(11)**				23.07	1.58	17.44	5.06	19.87	1.24	23.1	19.36
1	10.82	513-86-0	L-Lactic acid	0	0	0	0	2.2	0.16	0	0
2	20.61	79-31-2	2-Methyl-propanoic acid,	0	0	4.02	0.65	0	0	32.87	15.25
3	17.07	631-61-8	Acetoic acid	0	0	5.83	0.13	0	0	72.1	51.49
4	23.06	257932-17-5	6-Hydroxybicyclo[2.2.2]octane-2-carboxylic acid	0	0	2.77	0.92	0	0	25.5	18.86
5	23.67	503-74-2	Isovaleric acid	7.4	0.27	27.73	4.2	15.56	2.39	663.6373	88.33678
6	27.63	646-07-1	4-Methyl-pentanoic acid	0	0	0	0	0	0	31.72	21.6
7	28.59	142-62-1	Hexanoic acid	13.15	0.82	10.35	1.78	14.07	2.45	102.05	8.23
8	32.5	124-07-2	Octanoic acid	1.84	0.3	2.48	0.83	2.04	0.25	25.96	19.64
9	34.11	112-05-0	Nonanoic acid	2.71	0.42	1.22	0.41	2.96	0.34	0	0
10	37.69	65-85-0	Benzoic acid	1.5	0.12	0	0	2.34	0.28	0	0
11	38.86	57-10-3	Palmitic acid	4.28	0.23	5.68	1.89	4.57	0.08	18.41	6.41
**Esters(10)**											
1	0.87	105-54-4	Ethylbutyrate	3.28	0.33	5.14	0.93	5.25	0.84	11.05	1.48

2	5.06	539-82-2	Ethylvalerate	1.97	0.15	2.36	0.79	2.78	0.1	0	0
3	9.06	123-66-0	Ethylhexanoate	110.97	9.88	135.85	15.7	137.14	1.06	281.24	32.18
4	12.76	106-30-9	Ethylheptanoate	2.74	0.01	3.31	0.19	2.69	0.26	0	0
5	15.46	626-82-4	Butylhexanoate	1.84	0.12	0	0	2	0.19	0	0
6	16.21	106-32-1	Ethylcaprylate	10.54	0.66	18.92	1.43	10.56	0.65	43.39	18.01
7	21.35	100508-44-9	6,10,13-Trimethyltetradecyl isovalerate	0	0	3.99	1.33	0	0	8.93	0.94
8	21.63	6378-65-0	Hexylhexanoate	4.88	0.47	13.03	0.64	7.38	0.54	43.34	30.1
9	35.28	628-97-7	EthylHexadecanoate	3.16	0.26	4.66	0.1	4.81	1.27	16.97	6.54
10	38.98	544-35-4	linoleic acid ethyl ester	2.11	0.7	0	0	3.76	0.4	0	0
**Alkanes(6)**											
1	7.75	112-40-3	Dodecane	5.96	0.34	7.19	0.83	5.74	1.54	42.7	9.28
2	11.58	629-50-5	2-methyl-Decane	3.17	0.18	4.49	0.88	3.48	0.81	8	0.63
3	15.07	629-59-4	Hexadecane	5.95	0.1	6.05	0.39	6.39	0.11	15.63	7.23
4	18.31	31295-56-4	7-Methylpentadecane	4.1	0.46	6.88	0.3	5.62	0.83	8.29	0.9
5	20.09	2511-91-3	1-Cyclopropylpentane	5.07	0.35	2.97	0.99	6.78	0.05	0	0
6	20.36	32296-45-0	2,6,10-Trimethyl-dodecane	3.07	1.02	2.75	0.92	5.08	0.08	0	0
**Phenols(1)**											
1	34.55	7786-61-0	2-Methoxy-4-vinylphenol	2.92	0.15	3.28	1.09	6.24	2.53	33.75	12.77
**Furans (3)**											
1	23.28	98-00-0	3-Furanmethanol	0	0	15.67	2.66	0	0	195.05	48.46
2	24.91	22122-36-7	3-Methyl-2(5H)-Furanone	0	0	0	0	3.5	0.56	12.06	1.31
3	25.35	3857-25-8	5-Methyl-2-Furanmethanol	0	0	3.67	1.22	0	0	27.08	21.4
**Aromatic compounds(13)**											
1	18.87	100-52-7	Benzaldehyde	40.58	3.56	78.75	11.57	55.88	0.22	281.63	118.94
2	22.52	122-78-1	Benzeneacetaldehyd	10.45	1.3	14.54	0.27	13.25	1.63	193.79	132.07

3	22.67	98-86-2	Acetophenone	6.72	1.25	9.42	0.93	12.43	0.22	88.1	43.78
4	25.42	91-16-7	1,2-Dimethoxybenzene	2.56	0.02	4.59	0.26	4.86	0.29	0	0
5	25.53	121747-63-5	2,4,4-Trimethyl-3-(3-methylbutyl)cyclohex-2-enone	2.33	0.34	0	0	2.36	0.18	0	0
6	25.7	150-78-7	1,4-Dimethoxy-Benzene	1.3	0.43	0	0	2.68	0.25	0	0
7	27.01	101-97-3	Ethylphenylacetate	1.68	0.06	0	0	2.19	0.04	0	0
8	27.72	1445-91-6	a-Methyl-benzenemethanol	2.2	0.14	0	0	3.44	0.7	0	0
9	29.12	100-51-6	Benzyl alcohol	30.54	9.82	23.46	3.89	70.57	8.03	196.05	94.24
10	29.24	2021-28-5	Benzenepropanoic acid ethyl ester	9.25	0.55	21.51	0.31	10.91	0.78	60.88	36.25
11	29.8	60-12-8	Phenylethyl alcohol	165.96	4.69	200.62	27.32	253.42	22.45	1387.05	9.98
12	30.16	4701-36-4	1-Ethyl-1-propenylbenzene	0	0	4.51	0.35	0	0	48.12	25.72
13	32.09	6380-23-0	4-Ethenyl-1,2-dimethoxy-benzene	1.71	0.15	3.31	0.23	2.86	0.3	0	0
**Nitrogen compounds(14)**											
1	10.01	109-08-0	2-Methylpyrazine	3.39	0.85	5.87	1.6	4.63	0.33	19.81	10.11
2	12.04	123-32-0	2,5-Dimethylpyrazine	18.66	5.66	5.37	0.84	36.03	2.04	17.65	6.51
3	12.27	123-32-0	2,6-Dimethylpyrazine	7.26	0.02	11.49	2.05	11.01	0.23	36.19	7.09
4	12.89	5910-89-4	2,3-Dimethylpyrazine	0	0	22.28	4.41	15.76	2.01	92.65	56.17
5	14.29	13925-03-6	2-Ethyl-5-methyl-pyrazine	3.36	1.2	5.41	1.02	4.2	0.33	20.25	4.99
6	14.88	14667-55-1	Trimethylpyrazine	25.54	4.6	120.98	23.14	66.78	4.45	575.05	23.72
7	16.28	13925-07-0	2-Ethyl-3,5-dimethyl-Pyrazine	0	0	17.1	2.51	2.41	0.54	66.6	41.98
8	17.2	1124-11-4	Tetramethylpyrazine	124.84	18.55	1485.75	282.16	331.13	35.58	9247.78	645.43
9	17.77	13925-09-2	2-Ethenyl-6-methyl-Pyrazine	4.9	0.07	6.98	0.69	6.76	0.2	0	0

10	18.49	17398-16-2	2,3,5-Trimethyl-6-ethylpyrazine	0	0	9.98	2.18	4.1	1.46	71.58	67.9
11	20.29	23069-75-2	8-Methyl-1,2,4-Triazolo[4,3-b]pyridazine	1.54	0.11	4.7	0.94	3.04	0.34	0	0
12	30.95	1072-83-9	1-(1H-pyrrol-2-yl)-（2-Acetylpyrrole）-ethanone	4.38	0.72	10.86	2.08	4.7	0.24	82.5	45.56
13	31.89	1003-29-8	1H-Pyrrole-2-carboxaldehyde	0	0	0	0	0	0	94.95	65.54
14	37.87	120-72-9	Indole	1.62	0.26	0	0	0	0	0	0
**Sulfur compounds(3)**											
1	13.99	3658-80-8	Dimethyl trisulfide	0	0	0	0	0	0	7.7	0.82
2	25.5	7330-31-6	Dimethyl pentasulfide	0	0	0	0	0	0	11.52	1.32
3	33.2	54845-33-9	2,5-bis(2-methylpropyl)-Thiophene	5.07	0.43	5.18	0.33	5.87	0.3	0	0
**Others(8)**				5.07	0.43	5.18	0.33	5.87	0.3	19.22	2.14
1	20.86	118-65-0	Caryophyllene	2.9	0.02	2.83	0.94	2.96	0.39	17.85	5.13
2	27.72	17373-93-2	2-Methylbenzyl acetate	0	0	1.25	0.42	0	0	47.63	10.79
3	24.32	1142-85-4	a-Terpinyl isovalerate	0	0	2.87	1.1	0	0	0	0
4	30.52	95-16-9	Benzothiazole	2.42	0.32	0	0	2.34	0.23	0	0
5	31.89	104-61-0	Dihydro-5-pentyl-2(3H)-Furanone	6.88	0.66	14.94	2.62	7.16	0.33	0	0
6	33.11	10396-80-2	2,6-Di-tert-butyl-4-hydroxy-4-methylcyclohexa-2,5-dien-1-one	3.21	0.21	2.57	0.86	3.61	0.03	78.16	10.81
7	33.2	6421-88-1	2-(4-Aminophenoxy)-ethanol	0	0	0	0	0	0	18.79	10.37
8	35.11	1008-88-4	3-Phenylpyridine	1.43	0.23	0	0	0	0	0	0

Values represent means (n = 3); SD, the value of STDEV.
